# Supplementary material for: Understanding the long-term impact of flooding on the wellbeing of residents: A mixed methods study
Source: PLoS One. 2022 Sep 22;17(9):e0274890. doi: 10.1371/journal.pone.0274890 (PMC9499214; doi:10.1371/journal.pone.0274890)
Supplement: S1 File — (PDF) [file pone.0274890.s001.pdf]

Living with Water Survey

Page 1: Survey information sheet

Project title: Living with Water - a joint project between Yorkshire Water, Hull City Council, the East Riding of Yorkshire Council and the Environment Agency. The main objectives of Living with Water are:

1) Place: to make Hull and the East Riding a great maritime area to live in, work in and visit.

2) Resilient: to reduce flood risk and build awareness and resilience.

3) Sustainable: to enable environmentally sensitive growth, which enhances local communities.

We are a team of researchers from the University of Hull working on the Living With Water project. *We are inviting you to take part in a research study by sharing your views with us through a survey. Before you decide whether or not to take part, it is important for you to understand why the research is being done and what it will involve. Please take time to read the following information carefully.*

The survey is being conducted in three areas of Hull that have experienced flooding: Derringham; Newland Avenue and Beverley Road; and North Carr (North Bransholme). We are conducting the survey in September and October 2018 using a team of University of Hull researchers. We are knocking on doors and asking householders if they could answer some questions. Alternatively you can complete the survey online. The survey will take between 10 and 20 minutes to complete. The last day of the survey is 19 October. *If you are not sure if you live inside the three target areas, you used to live in these areas but have since moved out, or you would like to complete the survey anyway - please feel free to complete the survey.*

The survey asks questions about whether people experienced flooding in 2007 or 2013, whether people feel at risk of flooding, and also whether you have taken any measures, or know of any measures taken by the Living with Water partners, to reduce flooding.

The aim of the survey is to help the Living with Water partnership find out what people know about flooding now, so in the future they can measure if the project has improved people's awareness of flooding measures and made people feel safer. The information from this survey will help the Living with Water partners take account of local people's needs. If you want more information on Living with Water please visit the website www.livingwithwater.co.uk

Participation in this survey will be voluntary. All information from the survey will be treated in confidence and individuals will not be identified. We will not record the answers given against your address - only your postcode. We only ask for your contact details at the end of the survey if you want to participate in any follow-up research. In such cases, contact details will be kept separately from answers given and again no individuals will be identified.

If you have any concerns or questions about the survey, questions about Living with Water, or if you would like to find out what happens with the information - please contact: Sam Ramsden on sam.ramsden@hull.ac.uk, Lisa Jones on l.m.jones@hull.ac.uk or Florence Halstead on f.halstead@2013.hull.ac.uk

---

The survey has received ethical approval from the University of Hull. Should you have any concerns about the conduct of this research project please contact: Jo Hawksworth, The Research Office, Faculty of Arts Cultures and Education (FACE), University of Hull, Cottingham Road, Hull, HU6 7RX. tel. 01482 466658. Email: j.hawksworth@hull.ac.uk

Page 2: Some basic information about you and your property

1) Are you 18 or over? (you need to be 18 or over to take part in this survey) ✱ *Required*

- Yes
- No

2) What is your **full postcode**? (full postcode, but not house or flat number) ✱ *Required*

3) When did you move into this address?

- 2016 or later
- between 2014 and 2015
- between 2008 to 2013
- from 2007 or before

4) Your household - who do you live with? (tick all that apply)

- On your own
- With a partner, husband or wife
- With child/ children
- With relatives
- With older people (over 65)
- Shared house with other tenants or friends

How many people live at the property?

5) Do you own or rent your property?

- Own
- Rent

*If you **rent** your property*, who do you rent it from?

- [ ] Private Landlord
- [ ] Council
- [ ] Registered Social Landlord
- [ ] Sheltered Housing Provider
- [ ] Other

*If **other***, please specify:

____

6) What is your property type?

- ○ Terraced House
- ○ Semi-detached house
- ○ Detached house
- ○ Ground-floor flat
- ○ Upstairs flat
- ○ Bungalow
- ○ Other

*If **other***, please specify

____

Page 3: Were you affected by the 2007 floods?

7) Was your household (people living in your house) in any way affected by the 2007 floods? Affected would include witnessed, helped people, disrupted or flooded.

- ○ Yes
- ○ No (not affected)

*If you answered **No (not affected)** please go to Question 8 (Page 4)*

How was your household (people living in your house) affected by the 2007 floods? Please choose one from: 1) Exposed, 2) Disrupted or 3) Flooded.

- ○ Exposed (witnessed effects of flooding, but was not directly disrupted or flooded)
- ○ Disrupted (homes were not flooded but lives were disrupted by flooding)
- ○ Flooded (property was flooded)

If your property was **flooded**, was it at your current address?

- ○ Yes
- ○ No

*If **no***, what was the postcode of your old address?

____

How did the 2007 floods affect your household? (tick all that apply)

☐ Evacuated house	☐ Damaged house	☐ Flooded Garden
☐ Affected utilities (Gas, Electricity)	☐ Affected water supply	☐ Affected food supply
☐ Damaged car	☐ Disrupted work	☐ Disrupted school
☐ Disrupted childcare	☐ Disrupted healthcare	☐ Disrupted essential travel (e.g. to schools, work, health facilities)
☐ Affected health and wellbeing	☐ Affected financial situation	☐ Other

*If **other***, please specify:

____

If you had to **evacuate** your house, how long did you leave your house for?

- less than 1 week
- between 1 week and a month
- between 1 and 6 months
- between 6 months and a year
- over a year
- never returned

If it affected your (or a member of your household's) **health and wellbeing**, can you explain in more detail?

What was the worst part of the 2007 floods?

Did you receive a flood warning before the 2007 floods?

- Yes
- No

*If **yes***, was this flood warning useful?

Did you or your household receive any help during the 2007 floods?

- Yes
- No

*If **yes***, who did you receive help from? (tick all that apply)

- Police
- Fire Brigade
- Ambulance
- NHS
- your local council
- Yorkshire Water
- Environment Agency
- Insurers
- Work
- School
- Family, Friends or Neighbours
- Community group, charity, church
- Residents Association
- Landlord
- Flood Warden
- Other

*If **other***, who helped you?

What was the most effective help you received?

Do you think you should have received more help? What kind of help? Who from?

Did you or your household help other people during the 2007 floods?

- Yes
- No

*If **yes***, can you explain in more detail?

Did your household experience any positive effects from the 2007 floods? (please describe briefly below)

Page 4: Plans in place or changes after the 2007 floods

8) Did you or your household have any plans or preparations in place before the 2007 floods?

- Yes
- No

*If **yes***, could you give more detail on your preparations and whether they helped reduce the impacts of flooding?

9) Did you or your household make any changes as a result of the 2007 floods?

- Yes
- No

*If **yes***, could you give more detail on what you did?

Page 5: Were you affected by the 2013 floods?

10) Was your household (people living in your house) in any way affected by the 2013 floods? Affected would include witnessed, helped people, disrupted or flooded.

- ○ Yes
- ○ No (not affected)

*If you answered **No (not affected)** please go to Question 11 (Page 6)*

How was your household (people living in your house) affected by the 2013 floods? Please choose one from: 1) Exposed, 2) Disrupted or 3) Flooded.

- ○ Exposed (witnessed effects of flooding, but was not directly disrupted or flooded)
- ○ Disrupted (homes were not flooded but lives were disrupted by flooding)
- ○ Flooded (property was flooded)

How were the effects of the 2013 floods compared to the 2007 floods?

Please don't select more than 1 answer(s) per row.

	Much smaller	Smaller	Same	Worse
Scale of effects	☐	☐	☐	☐

If your property was **flooded** was it at your current address?

- ○ Yes
- ○ No

*If **no**,* what was the postcode of your old address?

How did the 2013 floods affect your household? (tick all that apply)

- ☐ Evacuated house
- ☐ Damaged house
- ☐ Flooded garden
- ☐ Affected utilities (Gas, Electricity)
- ☐ Affected water supply
- ☐ Affected food supply
- ☐ Damaged car
- ☐ Disrupted work
- ☐ Disrupted school
- ☐ Disrupted childcare
- ☐ Disrupted healthcare
- ☐ Disrupted essential travel (e.g. to schools, work, health facilities)
- ☐ Affected health and wellbeing
- ☐ Affected financial situation
- ☐ Other

*If **other**,* please specify:

If you had to **evacuate** your house, how long did you leave your house for?

- less than 1 week
- between 1 week and a month
- between 1 and 6 months
- between 6 months and a year
- over a year
- never returned

If it affected your (or a member of your household's) **health and wellbeing**, can you explain in more detail?

What was the worst part of the 2013 floods?

Did you receive a flood warning before the 2013 floods?

- Yes
- No

*If **yes***, was this flood warning useful?

Did you or your household receive any help during the 2013 floods?

- Yes
- No

*If **yes***, who did you receive help from? (tick all that apply)

- Police
- Fire Brigade
- Ambulance
- NHS
- your local council
- Yorkshire Water

- [ ] Environment Agency
- [ ] Insurers
- [ ] Work
- [ ] School
- [ ] Family, Friends or Neighbours
- [ ] Community group, charity, church
- [ ] Residents Association
- [ ] Landlord
- [ ] Flood Warden
- [ ] Other

*If **other***, who helped you?

What was the most effective help you received?

Do you think you should have received more help? What kind of help? Who from?

Did you or your household help other people during the 2013 floods?

- ( ) Yes
- ( ) No

*If **yes***, can you explain in more detail?

Did your household experience any positive effects from the 2013 floods? (please describe briefly below)

Page 6: Were people you know well affected by the 2007 and/ or 2013 floods?

11) Were people you know well (e.g. family, friends or work-mates) affected by the **2007** floods?

- Yes
- No

*If **yes***, can you explain how it affected them?

12) Were people you know well (e.g. family, friends or work-mates) affected by the **2013** floods?

- Yes
- No

*If **yes***, can you explain how it affected them?

Page 7: Your vulnerability to flooding and measures taken

13) How concerned are you about floods?

Please don't select more than 1 answer(s) per row.

	1	2	3	4	5	
Not at all	☐	☐	☐	☐	☐	Very concerned

Can you explain your answer?

How often do you think there could be flooding that could damage your house?

- ○ once in every 10 years
- ○ once in every 11 to 30 years
- ○ once in every 31 to 50 years
- ○ 51 years or more (once in a lifetime)

14) What measures have you or your household taken to reduce your risk to flooding? (tick all that apply)

- ☐ Improved household flood defences (could include barriers, flood proofing, improving drainage, or moving valuables to safe place)
- ☐ Checked my flood risk
- ☐ Signed up to Environment Agency flood warning
- ☐ Prepared a flood plan
- ☐ Part of a local community flood information group (e.g. flood action group, flood warden)
- ☐ Made sure insurance covers flooding
- ☐ Prepared emergency flood kit. (Could include: torch, batteries, portable radio, first aid kit, food items)
- ☐ No measures yet
- ☐ Other

*If **other***, please specify:

If you have improved your **household flood defences**, can you tick all the measures you have taken?

- ☐ Sandbags
- ☐ Temporary flood barriers
- ☐ Moved valuable items to a safe place (e.g. upstairs)
- ☐ Moved kitchen upstairs
- ☐ Flood proof doors and windows
- ☐ Exterior walls: Water proof sealant
- ☐ Floors: damp-proof membrane
- ☐ Flood proof air bricks, or covered air bricks
- ☐ Improved drainage from property
- ☐ Drains and pipes: non-return valves
- ☐ Water Butts
- ☐ Landscaping (divert water away from property)
- ☐ Planting, greenery
- ☐ No measures yet
- ☐ Other

*If **other***, please specify:

15) Have you or your household received any information about preparing for floods?

- ○ Yes
- ○ No
- ○ Not sure

If you have **received any information**, who was it from? (tick all that apply)

- ☐ your local council
- ☐ Yorkshire Water
- ☐ Environment Agency
- ☐ Schools
- ☐ NHS
- ☐ Insurers
- ☐ Community group, charity or church
- ☐ Workplace
- ☐ Flood Action Group
- ☐ National Flood Forum
- ☐ Friends, family or neighbours
- ☐ Other

*If **other***, please specify:

What has been the most useful information you have received?

What would be the most effective method of communication to give you information?

- ☐ Social media (e.g. Twitter, Facebook)
- ☐ TV (e.g. local news)
- ☐ Local Radio
- ☐ Local Newspaper
- ☐ Mobile (SMS/ Text)
- ☐ Phone call
- ☐ Email
- ☐ Leaflet
- ☐ Other

If ***other****,* please specify:

Is English the best language to communicate with all the members of your household?

- ○ Yes
- ○ No

If ***no***, which other language(s) would it be good to receive communication in?

16) If there is another flood, do you think you or your household will receive a warning in good time?

- ○ Yes
- ○ No

If ***yes***, who will you receive a flood warning from?

17) Have you (or a member of your household) attended any community-level flood awareness activities, events or meetings?

- ○ Yes
- ○ No

If ***yes****,* which community-level flood awareness activities, events or meetings have you attended? (tick all that apply)

- Through council, Environment Agency and/ or Yorkshire Water
- Flood Action Groups
- Community Flood Wardens
- Residents Association
- Through schools
- Through work
- Through community group, charity or church
- Other
- Not yet

*If **other***, can you describe in more detail?

Have you helped organise any of the above activities, meetings or events? If so, can you give more detail?

18) Do you know about any of the following measures that are working to reduce flooding in Hull (and surrounding areas of East Riding)?

Please don't select more than 1 answer(s) per row.

	Not heard of	Heard of but limited knowledge	Good understanding
Tidal barrier			
Flood defence walls (e.g. perspex barriers at Humber, Humber Defence, Hull Defence)			
Flood Alleviation Schemes (e.g. Willerby & Derringham - WADFAS, Cottingham & Orchard Park - COPFAS, Anlaby & East Ella - AEEFAS)			
SuDS (Sustainable Drainage Systems which slow flow of water into sewer systems, e.g. Rain Gardens or Rain Butts)			
Specific support for vulnerable properties (e.g. household flood defence measures)			
Lagoons & Aqua Greens			
Planting trees/ improving green spaces/ biodiversity/ green roofs			
Improvements to pumping stations			
Other			

*If **other***, please explain in more detail below:

Do you know if any of these measures are helping your local community?

- ◯ Yes
- ◯ No
- ◯ Not sure

Is there anything you don't like about any of these measures, or do you have any concerns? If so, can you describe briefly.

19) Who would you **ask for help** if there was a flood that looked like it could damage your house? (tick all that apply)

☐ Call 999	☐ Police	☐ Fire Brigade
☐ Ambulance	☐ your local council	☐ NHS
☐ Yorkshire Water	☐ Environment Agency	☐ Insurers
☐ Work	☐ School	☐ Family, Friends or Neighbours
☐ Community group, charity, church	☐ Residents Association	☐ Landlord
☐ Flood Warden	☐ No one	☐ Other

if other, who would you ask for help?

20) Who would you **report it to** if there was a flood that looked like it could damage your house? (tick all that apply)

☐ Call 999	☐ Police	☐ Fire Brigade
☐ Ambulance	☐ your local council	☐ NHS
☐ Yorkshire Water	☐ Environment Agency	☐ Insurers
☐ Work	☐ School	☐ Family, Friends or Neighbours
☐ Community group, charity, church	☐ Residents Association	☐ Landlord
☐ Flood Warden	☐ No one	☐ Other

*If **other,*** who would you report it to?

21) How well is your house protected against flooding? (Rank from 1 to 5)

Please don't select more than 1 answer(s) per row.

	1	2	3	4	5	
Very low protection	☐	☐	☐	☐	☐	Very high protection

22) How quickly do you think you would recover if your household was affected by flooding? (Rank from 1 to 5)

Please don't select more than 1 answer(s) per row.

	1	2	3	4	5	
Very slow recovery	☐	☐	☐	☐	☐	Very fast recovery

23) Do you think more should be done to reduce your household's risk of flooding?

- ○ Yes
- ○ No
- ○ Not sure

If ***yes***, what should be done and by who?

24) Thinking about your house or property, do you know what type of flooding your property is vulnerable to? (tick all that apply)

- ☐ None
- ☐ River (water course flooding) - e.g. from River Hull or Holderness Drain
- ☐ Surface water flooding (e.g. from intense rainfall that cannot drain away)
- ☐ Sewer flooding (if sewers are at capacity and water cannot drain away)
- ☐ Tidal/ Sea flooding (e.g. from Humber)
- ☐ Groundwater flooding (e.g. from a rise in the water table)
- ☐ Not sure

25) Do you think climate change has increased the risk of flooding in your area of Hull?

Page 8: Some final information about you

26) What is your age group?

- 18 to 24
- 25 to 34
- 35 to 50
- 51 to 64
- 65 to 79
- 80 plus

27) What is your gender?

28) What is your ethnic group? Choose one option that best describes your ethnic group or background

- English/Welsh/Scottish/Northern Irish/British
- Irish
- Gypsy or Irish Traveller
- Any other White background
- White and Black Caribbean
- White and Black African
- White and Asian
- Any other Mixed/Multiple ethnic background
- Indian
- Pakistani
- Bangladeshi
- Chinese
- Any other Asian background
- African
- Caribbean
- Any other Black/African/Caribbean background
- Arab
- Any other ethnic group

If you have anwered 'any other' white, mixed or multiple, asian, black or other ethnic groups, please describe below.

These categories have been taken from the Office of National Statistics: thttps://www.ons.gov.uk/methodology/classificationsandstandards/measuringequality/ethnicgroupnationalidentityandreligion [accessed 25th July 2018]

29) Are you working? (tick all that apply)

- Employed
- Self-employed
- Out of work
- Retired
- Student

- ☐ Caring for relatives
- ☐ Volunteering
- ☐ Other

If ***other***, please describe below

What is your usual occupation?

30) Do you consider yourself to have a disability?

- ○ Yes
- ○ No

31) Is there anything else you would like us to know about your experiences or feelings about flooding?

32) Just before you submit your survey responses and go onto the page with further information - would you be willing to answer some more questions in an interview at a later date?

- ○ Yes
- ○ No

If ***yes,*** then please leave your preferred method of contact here e.g. email address or phone number. *(This survey information will still remain confidential and anonymous - individual answers will not be identified).*

Page 9: Thank you

Thank you very much for completing this survey.

As described in the introduction, the aim of the survey is to produce a baseline so that the Living with Water partnership can monitor and evaluate whether their project has improved people's awareness of flooding measures and made people feel safer. The information from this survey could therefore help the Living with Water project take account of local people's needs.

All information will be confidential and anonymous.

If you want more information about Living with Water, you can visit the website: www.livingwithwater.co.uk - the website also enables you to contact the partners with questions, concerns or ideas.

There is also a Living with Water event in Queen's Gardens on the weekend of the 20th and 21st October. The event will inlcude the Hull-timate challenge, Humbrella Day Trail, and other family fun events, plus there will be information on the Living with Water project.

If you want to find out more about information, such as ways to help you plan for floods, reduce flood risk, concerns with insurance, or on the different flood alleviation measures in Hull and Haltemprice, you can contact the Hull City Council Flood RIsk Team: 01482 300 300 or email: flood.risk@hullcc.gov.uk

The East Riding of Yorkshire Council website also contain a range of information on local flood alleviation projects, including for Hull and Haltemprice: http://www2.eastriding.gov.uk/council/plans-and-policies/other-plans-and-policies-information/flood-risk/

You can sign up for Environment Agency flood warnings (and obtain more information on preparation) here: https://flood-warning-information.service.gov.uk/warnings

The National Flood Forum is a charity established to help, support and represent people at risk of flooding and can be contacted on 01299 403 055. Its website is: https://nationalfloodforum.org.uk/

Flood Re is a joint initiative between the Government and insurers. Its aim is to make the flood cover part of household insurance policies more affordable. You can find out more about Flood Re here - https://www.floodre.co.uk/

If you have any questions or concerns for the University of Hull about the survey, or would like further information, the contact details of the team are Sam Ramsden: sam.ramsden@hull.ac.uk or Lisa Jones: l.m.jones@hull.ac.uk, or you can contact the overall project manager - Professor Dan Parsons: d.parsons@hull.ac.uk or by phone on 01482 463012.
